# A flow cytometric method for characterization of circulating cell-derived microparticles in plasma

**DOI:** 10.3402/jev.v3.20795

**Published:** 2014-02-04

**Authors:** Morten Hjuler Nielsen, Henning Beck-Nielsen, Morten Nørgaard Andersen, Aase Handberg

**Affiliations:** 1 Danish PhD School of Molecular Metabolism, Faculty of Health Sciences, University of Southern Denmark, Odense, Denmark; 2 Department of Endocrinology M, Odense University Hospital, Odense, Denmark; 3 Department of Biomedicine, Aarhus University, Aarhus, Denmark; 4 Department of Clinical Biochemistry, Aarhus University Hospital, Aarhus, Denmark; 5 Department of Clinical Biochemistry, Aalborg University Hospital, Aalborg, Denmark

**Keywords:** extracellular vesicles, flow cytometry, coincidence occurrence, platelet-derived, monocyte-derived, endothelial cell-derived, erythrocyte-derived, tissue-factor, lactadherin

## Abstract

**Background and aim:**

Previous studies on circulating microparticles (MPs) indicate that the majority of MPs are of a size below the detection limit of most standard flow cytometers. The objective of the present study was to establish a method to analyze MP subpopulations above the threshold of detection of a new generation BD FACSAria™ III digital flow cytometer.

**Methods:**

We analyzed MP subpopulations in plasma from 24 healthy individuals (9 males and 15 females). MPs were identified according to their size (<1.0-µm), by Lactadherin-FITC labelling, and by exposure of cell-specific markers. The sensitivity of the flow cytometer was tested against that of a previous-generation instrument FC500. Reproducibility of the FACSAria and our set-up was investigated, and the percentage of phosphatidylserine (PS) exposing MPs binding Lactadherin was determined.

**Results:**

By using a flow cytometric approach we identified and quantitated MPs derived from platelets, monocytes, erythrocytes and endothelial cells. In addition, levels of tissue factor-positive MPs were determined. The FACSAria demonstrated improved sensitivity and increased MP detection range compared to the FC500 instrument. The reproducibility of PS+PMP and PS+MP measurements was 11.7 and 23.2%, respectively. When expressed as a percentage of total MPs, the PS-positive MP population represented 15.1±5.5%, and PS-positive MPs were significantly increased in men.

**Conclusion:**

We have established a method to measure MPs above the detection limit of a new generation flow cytometer and derived from a number of cell-types in a healthy population of men and women.

Microparticles (MPs) are small, membrane-bound vesicles of 0.1–1.0-µm diameter generated by budding or shedding from the plasma membrane and released by any cell type into the vascular compartment in response to activation or apoptosis (1).

Besides containing cytoplasmic components of the original cell and exposing proteins specific to the cells they derived from, MPs are believed to express a panel of negatively charged phospholipids, mainly phosphatidylserine (PS), which accounts for their procoagulant character and proinflammatory properties (2, 3).

In recent years, MPs have received special attention due to their potential involvement in inflammatory and autoimmune diseases, as well as in cardiovascular disorders and the metabolic syndrome (4, 5). In addition, MPs may be looked upon as biomarkers, providing information about their cellular origin as well as status of the parental cell.

Flow cytometry is an exceptional method to detect, quantify and characterize MPs in physiological and in pathological conditions. However, the small size and dim signals from most MPs challenge the sensitivity of flow cytometry. Several laboratories have reported that 0.5-µm is the cut-off value for accurately identifying MPs by using previous generation flow cytometers (6). However, the new generation of flow cytometers allow detection of MPs below this 0.5-µm limit (7–9), thereby providing access to measurement of MP subpopulations of smaller size (8–10).

The objective of the present study was to establish a method, using a new generation BD FACSAria™ III digital flow cytometer, to analyze MP subpopulations in plasma from healthy individuals.

## Materials and methods

### Study participants

Twenty-four healthy individuals (15 females and 9 males) were enrolled in the study.

All subjects (age >18 years and BMI<30) were apparently healthy as indicated by a medical questionnaire, and none had hypertension, a history of atherosclerotic disease, or were taking any medication. Blood samples were obtained in the fasting state, and the following parameters were determined in the routine laboratory: Platelet, leukocyte and monocyte counts, as well as haemoglobin levels were determined using a Sysmex XE-5000™ Automated Hematology System. A Cobas^®^ 6,000 analyzer (Roche) was used to determine concentrations of glucose, alanine transaminase (ALT), triglycerides, total cholesterol (Total-C), low-density lipoprotein cholesterol (LDL-C), high-density lipoprotein cholesterol (HDL-C) and apolipoprotein B (ApoB). LDL-C concentrations were estimated from the Friedewald formula. The local Scientific Ethics Committee approved the research protocol, and written informed consent was provided before enrolment.

### MP preparation and labelling

Blood samples for MP preparation were collected into sodium citrate anticoagulant at a 3.2% (0.105 M) final concentration and processed within 1 hour. Platelet-free plasma (PFP) was prepared at room temperature by serial centrifugations, (10 minutes at 1,800×g, 15 minutes at 3,000×g, and 5 minutes at 3,000×g), frozen in 1.5-ml tubes as 600 µL aliquots, and stored at −80°C until analysis.

Briefly, for each analysis 50 µL of freshly thawed PFP was transferred to a TruCount™ tube (BD Biosciences, New Jersey, USA) containing a lyophilized pellet, which releases a known number of fluorescent beads. Subsequently, MPs were labelled by adding 10 µL fluorescein isothiocyanate (FITC)-conjugated Lactadherin (83 µg mL^−1^, Haematologic Technologies Inc., Vermont, USA). Lactadherin is an opsonin released by stimulated macrophages and characterized by a PS-bonding motif (11, 12).

To identify the cellular origin of MPs, either of the following fluorescent mAbs to specific cell surface markers were added immediately after Lactadherin-FITC labelling: 8 µL Allophycocyanin (APC)-conjugated anti-human CD41, (6 µg mL^−1^ IgG1, κ (clone HIP8, Biolegend,San Diego, CA, USA)) and 5 µL peridinin chlorophyll protein complex with cyanin-5.5 (PerCP-Cy5.5)-conjugated anti-human CD14 (400 µg mL^−1^ IgG1, κ (clone HCD14, BioLegend)) were used to detect platelet- (PMP) and monocyte- (MMP) derived MPs, respectively. For the detection of erythrocyte-derived MPs (ErytMPs), 5 µL anti-human CD235a-APC (500 µg mL^−1^ IgG2b, κ (clone HIR2, eBioscience, San Diego, CA, USA)), was added. Endothelial cell-derived MPs were detected by a 2-color method using 8 µL APC-conjugated anti-human CD31 (50 µg mL^−1^ IgG1, κ (clone WM59, BioLegend)) and 3 µL PerCP-conjugated anti-human CD42b (400 µg mL^−1^ IgG1, κ (clone HIP1, BioLegend)). CD31 is expressed on both platelets and endothelial cells, whereas CD42b is restricted to platelets, allowing discrimination between PMPs and EMPs. Finally, 20 µL phycoerythrin (PE)-conjugated anti-human CD142 (12.5 µg mL^−1^ IgG1, κ (clone HTF-1, BD Pharmingen, New Jersey, USA)) was used to detect tissue factor-positive MPs. After 30 minutes of incubation (4°C, in the dark), 250 µL 0.22-µm filtered PBS was added to each labelled sample.

### Flow cytometry

Plasma samples were analyzed immediately after labelling using a BD FACSAria™ III High Speed Cell Sorter, which incorporates 3 air-cooled lasers at 488-, 633-, and 407-nm wavelengths, and equipped with BD FACSDiva™ software (v. 6.1.3). A 1.5 neutral density filter in front of the forward scatter detector was used to decrease the FSC signal and keep events above the established MP gate (see below) on scale. A Cytomics FC500 (Beckman Coulter) flow cytometer equipped with 2 laser lines (488 and 633 nm) was used for side-by-side comparison of MP measurements. Logarithmic amplification was used for all channels and isotype controls on plasma samples were used as negative controls.

**Fig. 1:**
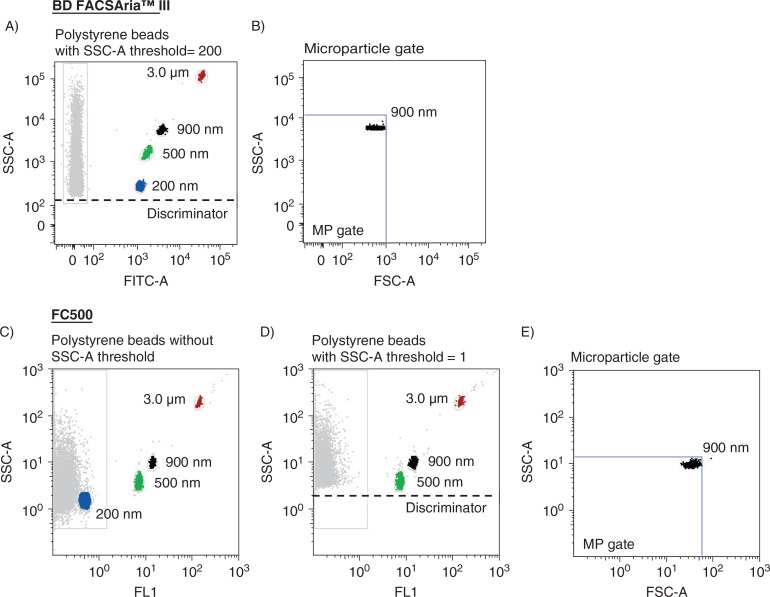
Construction of MP gates using size-calibrated fluorescent beads ranging from 200-nm to 3.0-µm. (A) Selection of bead subsets using the FACSAria instrument with a set SSC-A threshold of 200, and (B) construction of a MP gate with an upper limit of approximately 1 µm. (C) Selection of bead subsets using the FC500 instrument. (D) Threshold of 1 set on the SSC-A parameter eliminated the smallest (0.2 µm) beads. (E) Construction of a MP gate with an upper limit of approximately 1 µm. Background noise from the bead mix is shown in the left gate of the scatter plots.

A MP gate was established on the FACSAria instrument by preliminary standardization experiments using a blend of size-calibrated fluorescent beads, with sizes ranging from 0.2-µm (Invitrogen) to 3.0-µm (Megamix beads (0.5, 0.9 and 3.0-µm), Biocytex, Marseille, France) (Fig. 1A). A standardized calibrated-bead strategy using Megamix beads has previously been described (6, 8, 13–15). The upper and the outer limit of the MP gate was established just above the size distribution of the 0.9-µm beads in a forward (FSC-A) and side scatter (SSC-A) setting (log scale) using the “auto-gate” function inside the Flow Jo™ (v. 8.8.7, Tree Star, Inc., Oregon, USA) software (Fig. 1B). The lower limit was the noise threshold of the instrument, and an absolute minimum threshold of 200 was set at the SSC-A parameter (instead of FSC-A) to avoid exclusion of the smallest events. Similar strategy was used to establish a MP gate on the FC500 instruments (Fig. 1C–E), in which an absolute minimum threshold of 1 was set at the SSC-A parameter to limit the high background noise. This threshold excluded the 0.2-µm-sized beads (Fig. 1D). In order to separate true events from background noise, we defined MPs as particles that were less than 1.0 µm in diameter, had positive staining for Lactadherin, and expressed cell specific markers. Samples run on the FACSAria instrument were analyzed at rates below 10,000 events/seconds and stopped when 1,000,000 events within the MP gate were collected. Samples run on the FC500 instrument were analyzed at the lowest speed with a maximum acquisition rate of 3,300 events/seconds and stopped after a fixed time period of 120 seconds.

TruCount^TM^ beads (4.2-µm in diameter) with a known number of fluorescent beads were used for quantification following the manufacturer's instructions. In brief, events µL^−1^ plasma was calculated by the formula: (number of events in region containing MPs×number of beads per test)/(number of events in absolute count bead region×test volume). The number of beads per test-tube was provided by the manufacturer and the test volume was 50 µL. Data collection was not initialized until the count rate of the TruCount beads was stabilized. To examine contributions from background noise on each instrument, 0.22-μm-filtered PBS was analyzed under identical conditions (flow rate and time period) before each run.

### Side-by-side comparison and reproducibility testing

The sensitivities of the FACSAria and FC500 instruments were compared side-by-side by measuring MP levels in non-filtered and 0.22-µm-filtered plasma samples, and in plasma samples stained with FITC, PE or APC fluorochrome-conjugated antibodies. Reproducibility was tested on the FACSAria instrument only, by performing repeated measurements of absolute PS+ PMP and PS+ MP counts on aliquots of frozen PFP from all 24 study participants on 2 and 3 occasions, respectively, within 1 month. The coefficient of variation (CV, %) was calculated as SD/mean×100. Mean coefficient of variation was calculated as the square root of the mean of the squared CVs (Square root (($cv_{1}^{2}$)+($cv_{2}^{2}$)+($cv_{3}^{2}$)……($cv_{n}^{2}$))/n). Reproducibility testing on PMPs was chosen to compare results with findings in previous reproducibility studies on PMPs. Each flow cytometry protocol was calibrated with beads before data acquisition.

### Statistical analysis

Statistical analyses were carried out using the STATA 11.2 statistical program (StataCorp LP, Texas, USA). MP data were expressed as median (interquartile range) and analyzed using nonparametric Mann-Whitney U tests. Continuous values with a normal distribution were expressed as the mean±standard deviation and analyzed with Student's *t*-test. Differences were significant at p≤0.05.

## Results

### Side-by-side comparison

**Fig. 2:**
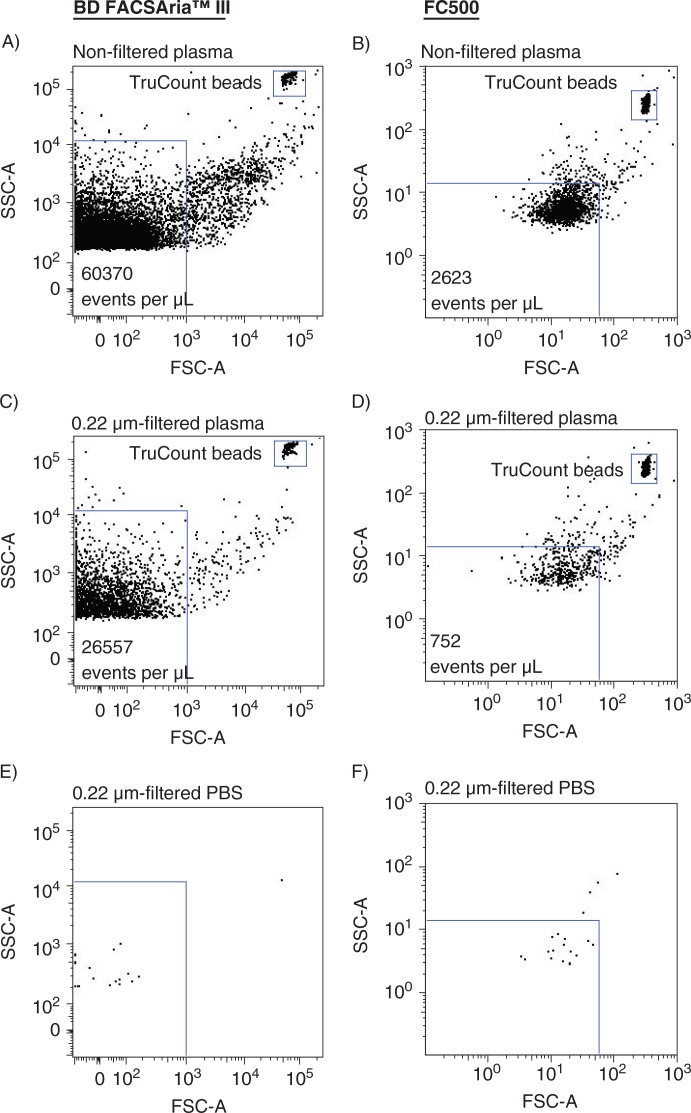
Side-by-side determination of absolute events within the MP gate using FACSAria (left panel) and FC500 (right panel) flow cytometers. (A,B) absolute events in non-filtered plasma. (C,D) absolute events in 0.22-µm-filtered plasma. TruCount beads are shown in the upper right corner. (E,F) evaluation of background noise contribution from 0.22-µm-filtered PBS analyzed under similar conditions (flow rate and time period).

**Fig. 3:**
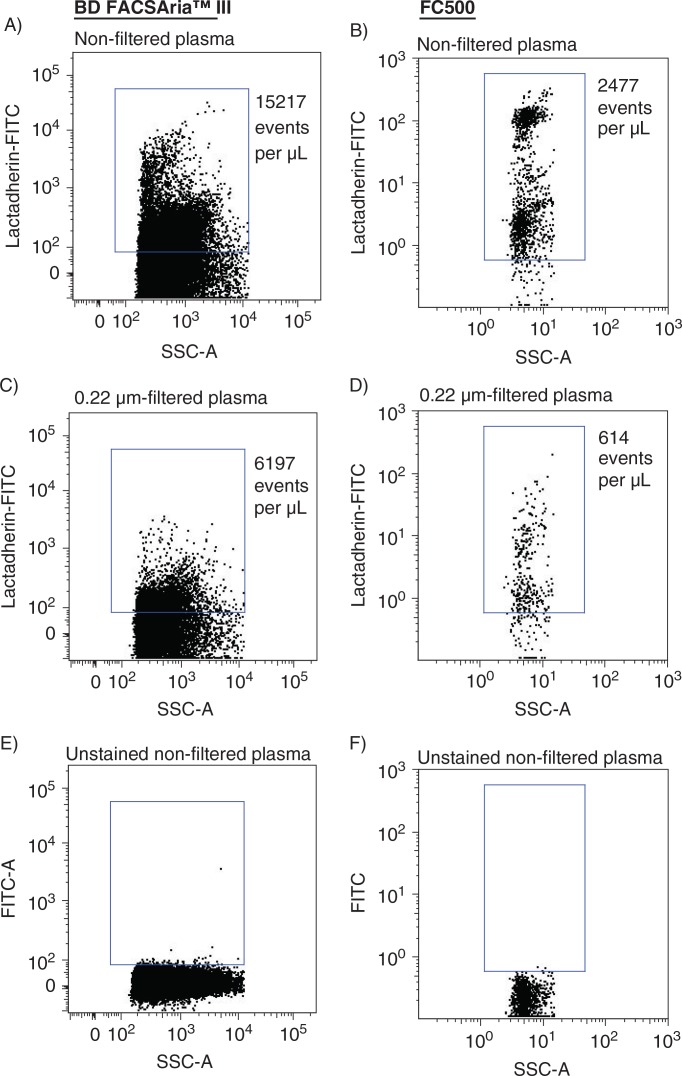
Side-by-side determination of Lactadherin-FITC positive events using FACSAria (left panel) and FC500 (right panel) flow cytometers. (A,B) Lactadherin-FITC positive events in non-filtered plasma. (C,D) Lactadherin-FITC positive events in 0.22-µm-filtered plasma (filtered prior to staining). (E,F) negative control for Lactadherin binding (unstained non-filtered plasma).

**Fig. 4:**
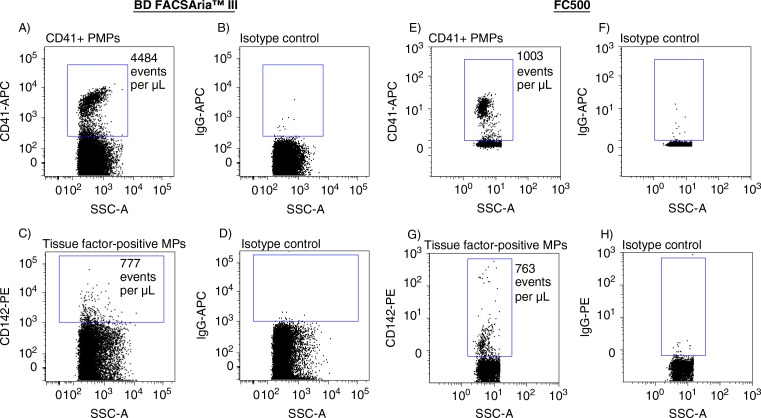
Side-by-side determination of (A,E) CD41 + PMPs and (C,G) CD142+ (tissue factor-positive) MPs using FACSAria (left panel) and FC500 (right panel) flow cytometers. Right columns correspond to the respective isotype controls.

The sensitivities of a new-generation FACSAria and a previous-generation instrument FC500 were compared side-by-side. Absolute events determined in non-filtered and in 0.22-µm-filtered plasma using both instruments are shown in Fig. 2. Analyzing non-filtered (Fig. 2A and 2B) and 0.22-µm-filtered (Fig. 2C and 2D) plasma on the FACSAria instrument resulted in a 23- and 35-fold, respectively, increase in events per µL plasma, compared to events encountered when analyzing identical samples on the FC500. Overall, filtration of the samples reduced the number of events by 56.0% (FACSAria) and 71.3% (FC500), respectively. Background noise contributions from the instruments and from 0.22-µm-filtered PBS, evaluated under similar conditions (flow rate and time period) were negligible (Fig. 2E and 2F). Events inside the MP gates were further specified by Lactadherin-FITC labelling to distinguish true events from electronic noise and thereby increase the specificity of MP detection. Figure 3 illustrates Lactadherin-FITC positive events in non-filtered and in 0.22-µm-filtered plasma when analyzed on both instruments. The number of Lactadherin-FITC positive events in non-filtered (Fig. 3A and 3B) and 0.22-µm-filtered (Fig. 3C and 3D) plasma was increased 6- and 10-fold, respectively, when measured on the FACSAria and compared to numbers determined using the FC500. Filtration of the samples prior to staining reduced Lactadherin-FITC positive events by 59.3% (FACSAria) and 75.2% (FC500), respectively. As a control for Lactadherin binding, we found negligible scatter signal in a parallel experiment on an unstained plasma sample, in which PBS was added instead of Lactadherin (Fig. 3D and 3F). Finally, the higher sensitivity (and the extended measureable size range) of the FACSAria instrument resulted in a 4-fold increase in the number of PMPs, when compared to measurement on the FC500 (Fig. 4A and 4E), whereas comparable numbers of CD142+MPs were detected on both instruments (Fig. 4C and 4G).

### Test for coincident occurrence

**Fig. 5:**
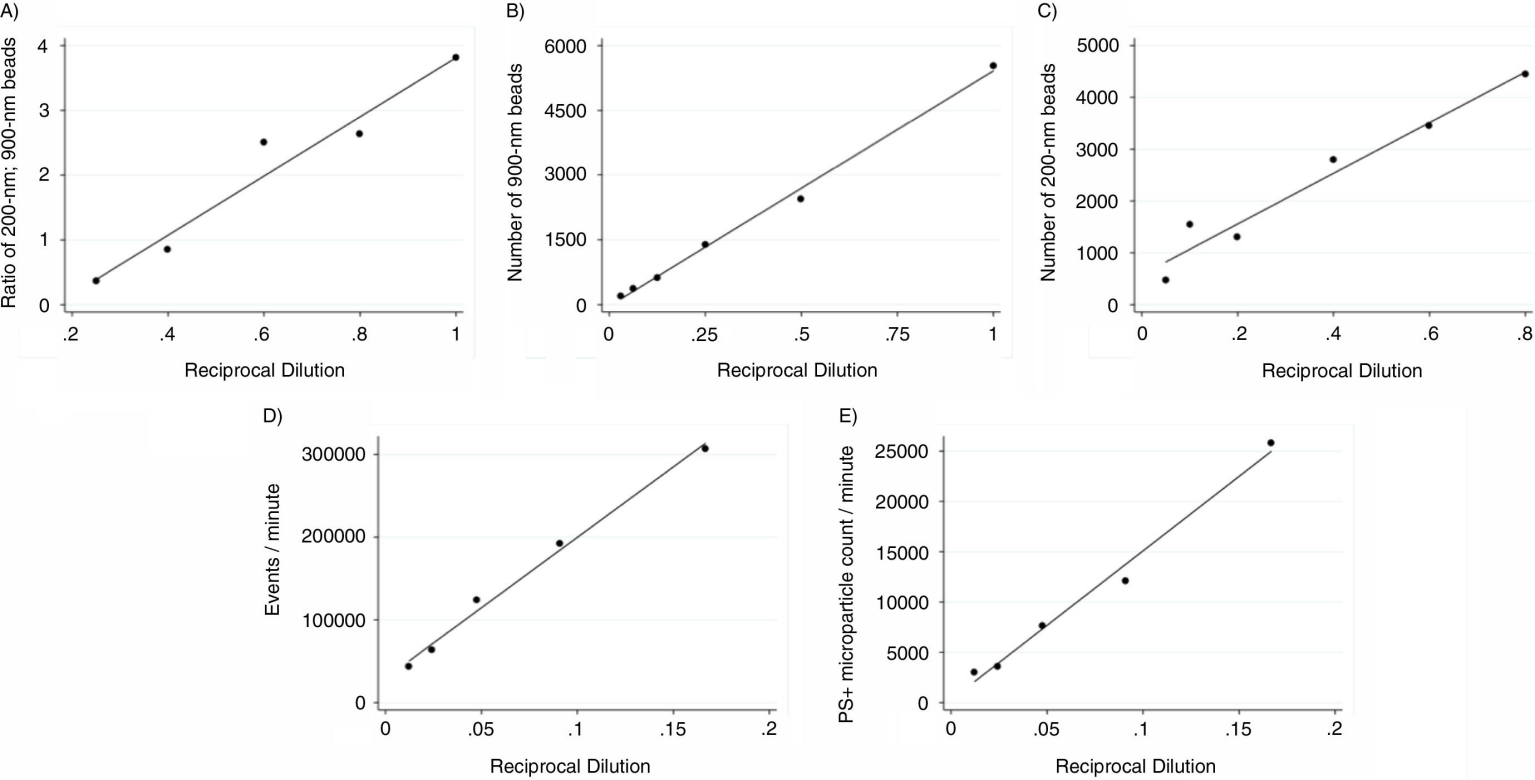
Test for coincidence detection. (A) Serial dilutions of 200-nm beads mixed with a fixed number of Megamix beads, using the 900-nm sized beads as reference. We collected events triggered on side scatter (SSC-A) with a threshold of 200. Expressed is the ratio of 200-nm beads vs. 900-nm beads at each dilution. R^2^=0.951, as determined by linear regression. (B–C) To support our findings we conducted serial dilutions of 900-nm beads (R^2^=0.995) and 200-nm beads (R^2^=0.955), respectively. By serial dilution of plasma samples we observed a proportionally drop of all events within (D) the MP gate (R^2^=0.991) and of (E) all PS-positive events within the MP gate (R^2^=0.987).

After confirming a high range of detection using the FACSAria instrument, we tested the instrument for coincidence detection of particles by measuring serial dilutions of 200-nm beads mixed with a fixed number of Megamix beads, using the 900-nm sized beads as reference. As shown in Fig. 5A, event rates dropped proportionally with sample dilution. Similar results were obtained when measuring serial dilutions of 900-nm (Megamix beads) and 200-nm beads separately, as illustrated in Fig. 5B and 5C, respectively. The concentration of nano-sized particles in human PFP is high compared to the class of particles, which has been the focus in the present study. We therefore sought to test for coincident occurrence by measuring serial dilutions of human plasma samples. As shown in Fig. 5D and 5E, respectively, event rates within the MP gate as well as events positive for PS dropped proportionally with sample dilution.

### Coefficient of variation

Reproducibility testing of our set-up was performed by measuring absolute PS+ PMP and PS+ MP counts in 2 and 3 independently repeated measures, respectively, over a 1-month period on the same instrument, resulting in coefficient of variations of 11.7 and 23.2%, respectively.

### MP subpopulations in healthy subjects

**Fig. 6:**
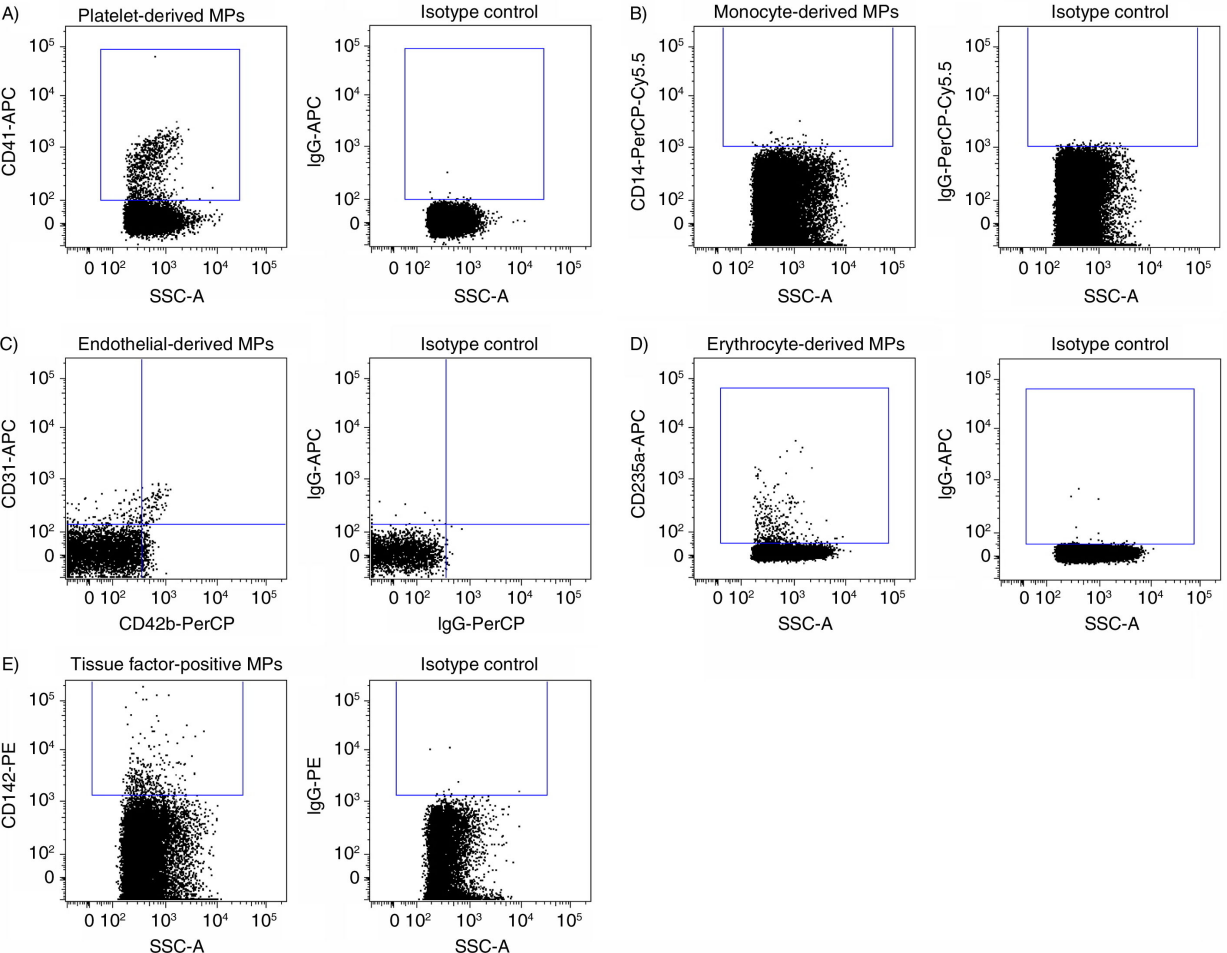
Detection of microparticle (MP) subpopulations in human plasma. (A) Platelet-derived MPs (CD41 + PMPs). (B) Monocyte-derived MPs (CD14 + MMPs). (C) Endothelial-derived microparticles (CD31+ /CD42b− EMPs). (D) Erythrocyte-derived microparticles (CD235a + ErytMPs). (E) Total tissue factor-positive microparticles (CD142+(TF) MPs). Right columns correspond to the respective isotype controls.

Mean age of women and men did not differ significantly, and routine laboratory measurements were all within age- and gender-specific reference intervals, with no gender difference except for increased haemoglobin (p<0.0001) and decreased HDL-C (p=0.0003) values in men (Table I).Using the above-defined MP gate and subsequent gating for Lactadherin-FITC binding events and by using specific markers, we detected MPs derived from platelets (CD41+), monocytes (CD14+), endothelial cells (CD31+/CD42b−) and erythrocytes (CD235a) (Fig. 6A–D). In addition, we identified MPs positive for tissue factor (CD142+) (Fig. 6E).

**Table I:** Characterization of study population

	Women (n=15)	Men (n=9)	p
Age (years)	49.8±9.3	42.7±9.7	0.088
BMI (kg/m^2^)	23.1±3.7	24.7±2.0	0.247
Systolic bp (mm Hg)	122.5±11.5	116.8±8.2	0.203
Diastolic bp (mm Hg)	75.3±6.4	73.4±6.9	0.520
Platelet count (10^9^/L)	247±56.8	220.9±35.3	0.225
Leukocyte count (10^9^/L)	5.0±1.4	5.1±1.0	0.880
Monocyte count (10^9^/L)	0.39±0.13	0.45±0.11	0.250
Haemoglobin (mmol/L)	8.35±0.36	9.27±0.30	<0.0001
Total-C (mmol/L)	5.28±0.58	4.78±0.66	0.065
LDL-C (mmol/L)	2.91±0.50	2.83±0.55	0.741
HDL-C (mmol/L)	1.91±0.32	1.38±0.22	0.0003
ApoB (g/L)	0.78±0.11	0.77±0.15	0.803
Glucose (mmol/L)	5.34±0.52	5.57±0.37	0.268
ALT (U/L)	19.3±10.8	22.6±7.4	0.431
TG (mmol/L)	1.0±0.3	1.3±0.7	0.270

P-values were determined by Student's *t*-test for continuous values with a normal distribution. Values are shown as the mean±standard deviation. Abbreviations: BMI, body mass index; ALT, alanine transaminase; TG, Triglycerides; ApoB, Apolipoprotein B; Total-C, total cholesterol; LDL-C, LDL cholesterol; HDL-C, HDL cholesterol; bp, blood pressure.

By assessing both PS-positive and PS-negative MPs, we observed a significantly higher amount of PS-negative MP counts compared to PS-positive MP counts, both when analyzing total MPs (p<0.0001) and TF+MPs (p<0.0001). In contrast, PS-positive MP counts were higher when analyzing PMPs (p<0.0001), MMPs (p<0.0408), EMPs (p<0.0001), except for ErytMPs, where equal amounts of PS-positive and PS-negative events were found. When expressed as a percentage of total MPs, the PS-positive MP population represented 15.1±5.5%, and of these 12.7±8.7% were derived from platelets, 1.0±0.6% from endothelial cells, 1.2±0.6% from erythrocytes, 0.03±0.02 from monocytes and 1.7±0.9% were exposing tissue factor. The mean (±SD) percentages of PS-positive events within each subpopulation were determined, and results are summarized in Table II.

**Table II:** PS-positive and PS-negative events within the MP gate

	PS-positive events	PS-negative events	% PS-positive events
Total events	15,711 (10,462–42649)	69,197 (47,126–310,839)	15±5
CD41+ (PMPs)	2,034 (1,419–2,777)	132 (87–228)	92±7
CD14+ (MMPs)	6 (3–10)	6 (4–16)	41±18
CD31+/CD42b− (EMPs)	179 (120–246)	39 (18–81)	74±19
CD235a+ (ErytMPs)	217 (152–293)	212 (115–322)	49±11
CD142+ (TF + MPs)	233 (205–403)	759 (558–2,070)	23±8

Values are shown as events µL^−1^ plasma and expressed as median (interquartile range). Abbreviations: MPs, microparticles; EMPs, endothelial microparticles; PMPs, platelet microparticles; MMPs, monocyte microparticles; ErytMPs, erythrocyte microparticles; TF, tissue factor; PS, phosphatidylserine.

### Gender-specific differences in circulating MPs

Using this flow cytometric approach, we observed increased levels of MPs in men, compared to women (Table III). Focusing on all PS-positive events only, significant differences were found in total PS+ events (p=0.025), PS+ MMPs (p=0.018) and PS+ TF+ MPs (p=0.013). Interestingly, men had significantly lower percentages of PS-positive MPs, except for MMPs and ErytMPs, when compared to women.

**Table III:** Gender-specific differences in circulating MPs

	Women (n=15)	Male (n=9)	p
All events	73,101 (54,725–130,676)	648,561 (90,952–1,138,487)	p=0.019
PS+ events	12,463 (9,236–17,543)	41,538 (18,825–70,659)	p=0.025
% PS+ events	17.3±3.5	11.2±6.2	p=0.005
All PMPs	2,007 (1,428–3,020)	2,530 (2,151–3,450)	p=0.222
PS+ PMPs	1,835 (1,384–2,876)	2,137 (2,020–2,475)	p=0.493
% PS+ PMPs	94.2±2.1	87.9±10.1	p=0.027
All MMPs	9 (7–13)	33 (10–46)	p=0.029
PS+ MMPs	4 (2–6)	11 (7–12)	p=0.018
% PS+ MMPs	42.7±14.8	39.2±23.6	p=0.657
All EMPs	175 (139–257)	418 (235–541)	p=0.016
PS+ EMPs	149 (119–218)	254 (202–297)	p=0.069
% PS+ EMPs	82.8±11.1	60.4±21.2	p=0.003
All ErytMPs	374 (189–516)	556 (342–1,845)	p=0.089
PS+ ErytMPs	202 (101–269)	251 (179–721)	p=0.069
% PS+ ErytMPs	50.4±9.3	47.0±12.8	p=0.459
All TF+MPs	829 (697–1,385)	3,453 (1,003–5872)	p=0.010
PS+ TF+MPs	209 (199–308)	396 (225–531)	p=0.013
% PS+ TF+MPs	26.1±6.4	17.0±8.7	p=0.007

Values are shown as events µL^−1^ plasma and expressed as median (interquartile range). Abbreviations: PS, phosphatidylserine; MPs, microparticles; EMPs, endothelial microparticles; PMPs, platelet microparticles; MMPs, monocyte microparticles; ErytMPs, erythrocyte microparticles; TF, tissue factor.

## Discussion

Previous studies on MP sizing indicate that the majority of MPs are below the detection limit of most standard flow cytometers (9, 16–18). In the present study, we describe a quantitative flow cytometric approach to determine MP subpopulations in plasma with a diameter below 1 µm and extending down to the noise threshold of a FACSAria^TM^ III flow cytometer. By gating for Lactadherin-FITC binding events and for specific cellular markers, we confirmed findings of others (9, 13, 19, 20) that MPs are derived from platelets (CD41+), monocytes (CD14+), endothelial cells (CD31+/CD42b−) and erythrocytes (CD235a). In addition, we identified MPs positive for tissue factor (CD142+).

To diminish background noise and to compare the sensitivities of the FACSAria and the FC500 instruments, a minimum threshold was set on the SSC parameter on both instruments. Although the use of polystyrene beads remains an imperfect model for general size calibration (8), using this threshold on the FC500 led to the disappearance of 200 nm beads, while still present on the FACSAria. The impact of this improved sensitivity was then evaluated side-by-side on PFP, either non-filtered, 0.22-µm-filtered or stained with fluorescence antibodies. Introducing a filtration step prior to staining and analysis had the highest impact on MP enumeration when analyzed on the FC500. Although such an inconsistency may alone be caused by a difference in SSC threshold settings, it is more likely a result of lower sensitivity. Interestingly, filtration removed approximately 60% of all PS-positive particles detected by the FACSAria indicating that a significant amount of particles passed through the filter and that the smallest MP detected by this kind of protocol display a size below 0.22 µm. This observation is in line with a previous study on plasma filtration and chromogenic capture of PS containing MPs (21).

The inability of the FC500 to separate particles below 0.5 µm in diameter was previously described (6, 8). Thus, gaining access to particles below the 500 nm-limit resulted in a 6-fold increase in MPs enumerated, and a 4-fold increase in PMP detection, while TF+ MP numbers were comparable. The present finding of increased numbers of PMPs when using an extended MP detection range is in line with previous observations (9). TF+ MP numbers were strikingly equal on both instruments, thus we speculate that the majority of tissue factor-bearing MPs are within the detection range of the FC500.

The ability of the FACSAria to detect MPs in the range of 0.3–1.0 µm has previously been demonstrated (10). Although our reproducibility testing was based upon 2 measures only, we observed good reproducibility of our PS+ PMP measurements, which were in line with findings from previous studies on PMPs (6, 22). To the best of our knowledge, we are the first to report reproducibility on absolute PS+ MP count in this size range, which we observed was higher than the CV of PS+ PMP measurements. The low day-to-day variation of the noise contribution did not cause this increase in CV value. The higher variation in PS+ MP counts may reflect small discrepancies in individual gating on the days of analysis. Future studies regarding standardization of gating strategies may improve reproducibility of PS+ MP determination.

Approximately 15% of all events within our size-defined MP gate were stained with Lactadherin-FITC, thus in agreement with results obtained in a recent study using an improved resolution flow cytometer (Apogee A40; Apogee System, Hemel Hempstead, UK), even though a slightly different MP size calibration standard was used (8). In that study, in which plasma samples were obtained from patients suffering from trauma, infection, vascular disease, renal insufficiency and venous thrombosis, Chandler and co-workers reported that, on average, 75% of Annexin V positive events were platelet-derived, whereas the present study, based on plasma samples from apparently healthy participants, report that less than 13% originates from platelets. Nevertheless, our results are in agreement with results obtained in a recent study on MPs from resting platelets (23). The same study demonstrated that the percentage of PS-positive events was highly depended upon the source of stimulation. Taken into account that the present study was based upon a healthy population, this may be an appropriate explanation for the above discrepancy on PS-positive PMP counts. Yet, another explanation for the observed dissimilarities may be found in the choice of MP marker. In the present study, PS-positive MPs were detected by using Lactadherin conjugated to FITC. This novel protein has demonstrated higher affinity than Annexin V for PS (about 2-fold), and has the potential advantage of non-calcium-dependent binding to PS (10, 24, 25). The fact that Lactadherin preferentially binds to highly curved membranes (11), suggest that more (and smaller) MPs are detected using Lactadherin, thereby potentially lowering the percentage of PS-positive PMPs, if compared against an increased total amount of PS-positive MPs.

Several studies have reported on MPs lacking PS expression (10, 19, 23, 26–29). Thus, the general understanding that all MPs express PS may be questioned. Indeed, a substantial portion of the analyzed MPs presented herein were PS negative. Although the biologic relevance of this finding is uncertain, it is currently believed that the negatively charged PS exposed on MPs act as a catalytic site for factor Xa and thrombin formation, giving MPs a procoagulant activity (21, 23, 30). Thus, a higher percentage of PS-positive MP counts may represent a more thrombotic state.

Our results indicate that PS expression levels vary within MP subpopulations, with PMPs displaying the highest percentages of PS-positive MPs. When comparing men and women, we observed a tendency towards a gender-specific difference in PS-positive MP counts, where men were found to encompass higher levels of PS-positive MP counts in general, but overall lower percentages of PS-positive events, as compared to women.

The majority of the female participants in our study group could be considered perimenopausal, and the fact that the incidence of stroke in women is known to increase substantially after menopause (31), may hypothetically be linked to the higher percentages of PS-positive MPs found in women, as shown in a recent study (32).

We observed gender-specific differences in circulating MPs with increased MP numbers in men, as compared with women. Our observations are in disagreement with previous studies reporting higher levels of total MPs, as well as MP subpopulations in women compared to men, which seems to be a menstrual cycle-specific difference (6, 33). The fact that the majority of the female participants in our study group are considered perimenopausal, and that our male participants are slightly younger compared to the female participants, may be a potential explanation for such a discrepancy. Most importantly, by using a more potent MP marker, and a more sensitive flow cytometer we expanded the detection range also to include MPs below the 500 nm limit.

Coincidence occurrence of multiple particles in the probe volume at the same time, also known as swarm detection, seems to be a general phenomenon in flow cytometry, especially when analyzing nanoparticles (34, 35). Cellular vesicles in a size range of 70–120 nm are present in a concentration of 0.5–5.0×10^10^ particles per mL (36). Thus, the concentration of small-sized particles is orders of magnitude higher than the class of particles, which has been the focus in the present study. We therefore sought to test for coincident occurrence by measuring serial dilutions of human plasma samples. We found that coincident occurrence, whether it is caused by small MPs or lipoprotein particles, can be neglected when analyzing at low flow rate (below 10,000 events per second). In addition, by defining MPs as particles with at diameter <1 µm, positive for PS and of specific cellular markers, we can increase the specificity of our flow set-up.

This study has some limitations. It has previously been reported that FSC signals of small particles are not only influenced by particle size but also by their refractive index, surface roughness, shape and possible light absorption (35, 37). Thus, FSC signals can only be used for approximate and relative sizing of small MPs. In the present study, we use 900-nm polystyrene microspheres from Megamix to define the upper limit of a forward scatter MP size gate. Although this gating strategy has been published by the ISTH Scientific Standardization Committee (14, 37), the difference in refractive index and thus forward angle light scatter between polystyrene beads and cellular MPs, as well as choice of reference materials to standardize MP detection, must be taken into account (8, 38). The flow cytometric method presented herein only allows analysis of MPs above the threshold of detection of the instrument, and potentially excludes smaller particles below this threshold. Thus, whether these analyzed MPs are representative or not of the total range of particles in human plasma remains unclear. In addition, although gating was set using the respective isotype controls we cannot exclude a minor contamination of PMPs (CD31+/CD42b+) in the gating of EMPs (CD31+/CD42b−).

Human PFP from normal individuals typically contains lipoprotein particles present in concentrations outnumbering extracellular vesicles and of similar size (36). Thus, we cannot eliminate the possibility that lipoprotein particles may contribute to scatter as well, although we measured on fasting samples and thus eliminated possible interference of chylomicrons. Finally, the number of individuals included in this study was only 24 (15 females and 9 males) and we did not take into account other demographic factors, which could influence MP levels. Clearly, these measurements need to be extended to larger and broader populations in order to establish a reference interval.

## Conclusions

In this study, we have established a method to measure plasma MPs derived from platelets, monocytes, erythrocytes, endothelial cells as well as tissue factor-positive MPs in a healthy population. Coincident occurrence is of minor significance when analyzing at a low flow rate. MP counts were higher in men compared to women, and the finding of gender-specific difference in PS exposure on MPs emphasizes that gender differences should be considered in study design.
